# Editorial: Walking, cycling and active travel as part of physical activity and public health systems

**DOI:** 10.3389/fspor.2023.1321450

**Published:** 2023-11-01

**Authors:** Paul Kelly, Jessica Bourne, Justin Richards, Deborah Salvo, Jason M. R. Gill

**Affiliations:** ^1^ Institute of Sport, Physical Education and Health Sciences, University of Edinburgh, Edinburgh, United Kingdom; ^2^ School of Health and Exercise Sciences, University of British Columbia, Vancouver, BC, Canada; ^3^ Te Hau Kori—the Centre for Physical Activity and Wellbeing, Victoria University of Wellington, Wellington, New Zealand; ^4^ Prevention Research Centre, Washington University in St. Louis, St. Louis, MO, United States; ^5^ School of Cardiovascular and Metabolic Health, University of Glasgow, Glasgow, United Kingdom

**Keywords:** walking, cycling, health, active travel, public health

**Editorial on the Research Topic** Walking, cycling and active travel as part of physical activity and public health systems

## Introduction

Increasing active travel is seen as a priority in many parts of the world, as it can facilitate mobility (e.g., reduced congestion), benefit the environment (e.g., reduced pollution and carbon emissions) and improve physical and mental health (1–3). The present Research Topic includes papers on several of the factors which we need to address to increase active travel.

## Establishing the benefits and known barriers

In a comprehensive review of the evidence, Logan et al. report the health benefits of cycling, the economic benefits, known barriers, and a summary of approaches to try and promote cycling. This can help make the case for cycling, support advocacy, and inform intervention development. Meanwhile, Kardan et al. reviewed the evidence related to cycling focussing on older adults, highlighting the importance of traffic safety in the evidence base.

In a study that focussed on health, Ding et al. reported positive associations with both physical and mental health and cycling to school in Chinese adolescents. In their multi-country study, Cordovil et al. showed that age of learning to cycle (necessary first step to getting people to cycle more) varies between countries and is likely impacted by individual, environmental, and temporal factors.

## Improving our measures

Several studies in this Research Topic investigated ways of measuring active travel, seen as a key step in facilitating behaviour change (4). Malnes et al. reported findings on the convergent validity of a new travel diary for school travel. The role of technology was also explored. Saito et al. assessed the validity and reliability of a smartphone application for measuring walking ability in older adults, while Pesola et al. looked at the ability of a thigh worn accelerometer to assess free-living cycling in children.

## The growth of e-cycling

Building on ever improving technology, sales of e-bikes have increased substantially in recent years and e-cycling is becoming increasingly prevalent. In their systematic review, Riiser et al. report evidence of health benefits from e-cycling, particularly increases in cardiorespiratory fitness. However the authors cautioned that the quality of these studies was generally low and more higher quality studies are required to determine the impact of e-cycling on health and the environment to support policy initiatives. Using a qualitative approach, Bourne et al. reported the determinants of e-cycling in people with Type 2 Diabetes. The enjoyment experienced while e-cycling was a key facilitator of engagement. The authors highlight that bike training was important to increase actual and perceived ability to e-cycle. These studies provide important factors for consideration in future e-cycling promotion efforts.

## Intervention development

Connell et al. reported the comprehensive development and piloting of a multi-component workplace cycling intervention targeting several identified barriers to cycling reported through engagement with the target population. This type of evidence based, multi-component intervention provides a helpful template for future initiatives to increase the potential for behaviour change.

## Policy and partnerships

Niven et al. described learning from 10 years of delivery organisations, academic researchers, and other stakeholders working in partnership on national walking promotion. Power et al. used a systems lens to understand walking policy at a country level. Moving from country to city level, Corr et al. used systems approaches to understand and develop cycling promotion strategies. Collectively these papers demonstrate that although building trusting cross-sectoral relationships can require significant investments of time this multidisciplinary collaboration seems necessary to promote cohesive action. Finally, Kahlmeier et al. described how health economic tools can be used to inform, influence and evaluate active travel intervention and policy. A tool such as the Health Economic Assessment Tool (HEAT) for walking and cycling is appealing to multiple stakeholders including academics, governments and private organisations.

## Looking forward

Considering the evidence included in this Research Topic and reflecting on the global impact of the COVID-19 pandemic over the last few years, it appears that we are at a critical juncture in the promotion of active transport. We have seen significant changes in worldwide mobility and travel behaviour. On one side there was a shift towards remote working environments, which reduced overall population commuting with more people working from home. Shifting commuters from motorised to active transport modalities has historically been a key target for walking and cycling initiatives as demonstrated by several studies in this Research Topic. Consequently, we may need to think more broadly about the types of “trips” we target in active transport advocacy.

Conversely, during the pandemic we saw some temporary shifts in the way people interacted with the physical environment immediately around their homes. Many people walked and cycled in their local neighbourhood and discovered local options to be physically active that they were not previously aware of or did not perceive to be safe. This may be something that is still fresh in the minds of the collective population and may have generated a latent demand for intervention, which may present a “once-in-a-generation” opportunity that must be acted upon to encourage lasting change, before it is too late. Several articles in this Research Topic outline how governments and organisations could intervene at the policy and community level to bring about more sustained active transport behaviour change.
